# Perioperative Management With Efanesoctocog Alfa in Patients With Haemophilia A in the Phase 3 XTEND‐1 and XTEND‐Kids Studies

**DOI:** 10.1111/hae.70017

**Published:** 2025-03-18

**Authors:** Robert Klamroth, Annette von Drygalski, Cedric Hermans, Young‐Shil Park, Anthony K. C. Chan, Alphan Kupesiz, María Teresa Alvarez‐Román, Lynn Malec, Elena Santagostino, Graham Neill, Linda Bystrická, Jennifer Dumont, Lydia Abad‐Franch, Lila‐Sabrina Fetita, Liane Khoo

**Affiliations:** ^1^ Vivantes Klinikum, Friedrichshain Berlin, and Institute of Experimental Hematology and Transfusion Medicine University Hospital Bonn Medical Faculty University of Bonn Bonn Germany; ^2^ Department of Medicine Division of Hematology/Oncology University of California San Diego San Diego California USA; ^3^ Haemostasis and Thrombosis Unit Division of Haematology Cliniques Universitaires Saint‐Luc Université Catholique de Louvain Brussels Belgium; ^4^ Department of Pediatrics Division of Hematology/Oncology Kyung Hee University Hospital at Gangdong Seoul South Korea; ^5^ McMaster Children's Hospital McMaster University Hamilton Canada; ^6^ Akdeniz University Antalya Turkey; ^7^ Hematology Department Hospital La Paz Madrid Spain; ^8^ Versiti Blood Research Institute Milwaukee Wisconsin USA; ^9^ Departments of Medicine and Pediatrics Division of Hematology and Oncology Medical College of Wisconsin Milwaukee Wisconsin USA; ^10^ Sobi Basel Switzerland; ^11^ Sanofi Reading UK; ^12^ Sanofi Cambridge Massachusetts USA; ^13^ Sanofi Paris France; ^14^ Institute of Haematology Royal Prince Alfred Hospital Sydney NSW Australia

**Keywords:** adults, BIVV001, children, haematology, haemophilia, haemostasis, surgical procedures, operative

## Abstract

**Introduction:**

The Phase 3 studies, XTEND‐1 (NCT04161495) and XTEND‐Kids (NCT04759131), showed once‐weekly efanesoctocog alfa provided high‐sustained factor VIII (FVIII) activity levels that translated into highly effective bleed prevention in patients with severe haemophilia A.

**Aim:**

This analysis evaluated the efficacy and safety of efanesoctocog alfa for perioperative management during XTEND‐1 and XTEND‐Kids.

**Methods:**

Patients undergoing major or minor surgery were to receive a single preoperative 50 IU/kg dose, with additional 30 or 50 IU/kg doses every 2–3 days as needed following major surgery. Outcomes assessed included FVIII activity levels, number and dose of efanesoctocog alfa injections, surgeon's/investigator's assessment of haemostatic response, total factor consumption, estimated blood loss, number and type of blood transfusions, and safety.

**Results:**

In XTEND‐1, 11 adults/adolescents underwent 12 evaluable major surgeries (6 orthopaedic). Eleven surgeries had one preoperative dose (median [range]: 49.9 [13–52] IU/kg); one had no preoperative dose. Median (range) total consumption from Day −1 to 14 was 163.3 (45–361) IU/kg. In XTEND‐Kids, two children underwent major surgery with a single preoperative loading dose (60.4 and 61.9 IU/kg). Across trials, 15 adults/adolescents underwent 18 minor surgeries and 8 children underwent 9 minor surgeries, with a single preoperative dose or no preoperative dose (5 surgeries in adults/adolescents). Haemostatic response was rated excellent for all surgeries. No surgeries required blood transfusion. No safety concerns or inhibitor development was reported.

**Conclusion:**

Efanesoctocog alfa provided highly effective perioperative protection in patients with severe haemophilia A.

**Trial Registration**: XTEND‐1: NCT04161495 https://clinicaltrials.gov/study/NCT04161495; XTEND‐Kids: NCT04759131 https://clinicaltrials.gov/study/NCT04759131

## 1 Introduction

Surgery poses a high bleed risk to patients with haemophilia A. Perioperative management requires the use of factor replacement therapy to maintain factor activity within target ranges recommended by the World Federation of Hemophilia (WFH) [1]. Monitoring factor VIII (FVIII) levels perioperatively is a burden for patients and hospitals. Efanesoctocog alfa is a first‐in‐class FVIII replacement therapy designed to provide high‐sustained factor activity levels by decoupling recombinant FVIII (rFVIII) from endogenous von Willebrand factor (VWF) to overcome the VWF‐imposed half‐life ceiling [2, 3, 4, 5]. A Phase 1 study (NCT05042440; EudraCT 2021‐000228‐37) of efanesoctocog alfa, a standard half‐life (SHL) rFVIII, and an extended half‐life (EHL) rFVIII in adults with severe haemophilia A found that efanesoctocog alfa had a three‐ to four‐fold longer elimination half‐life and three‐ to six‐fold greater exposure (area under the curve [AUC]) than EHL and SHL rFVIII, respectively [5]. Pharmacokinetic variability was also considerably lower with efanesoctocog alfa than with the other two rFVIII products [5].

The Phase 3 study, XTEND‐1 (NCT04161495), showed once‐weekly efanesoctocog alfa (50 IU/kg) prophylaxis provided mean FVIII activity levels >40 IU/dL for ∼4 days and 15 IU/dL at Day 7 in adults/adolescents with severe haemophilia A [6]. Efanesoctocog alfa prophylaxis provided superior bleed protection versus prior standard‐of‐care FVIII prophylaxis [6]. XTEND‐Kids (NCT04759131) showed once‐weekly efanesoctocog alfa prophylaxis provided mean FVIII activity >40 IU/dL for 3 days and >10 IU/dL for ∼7 days, leading to effective bleed prevention [7]. Efanesoctocog alfa has been approved for use in adults and children with haemophilia A for routine prophylaxis to reduce the frequency of bleeding episodes; on‐demand treatment and control of bleeding episodes; and perioperative management [8]. The objective of this analysis was to evaluate the efficacy and safety of efanesoctocog alfa for perioperative management in adults/adolescents and children who underwent major and minor surgeries during XTEND‐1 and XTEND‐Kids.

## 2 Materials and Methods

### 2.1 XTEND‐1 and XTEND‐Kids Study Designs

The XTEND‐1 and XTEND‐Kids study designs have been described previously [6, 7]. XTEND‐1 (NCT04161495) included adult/adolescent patients (≥12 years) with severe haemophilia A (<1 IU/dL [<1%] endogenous FVIII activity or a documented genotype that produces severe haemophilia A), previously treated with any recombinant and/or plasma‐derived FVIII, or cryoprecipitate for ≥150 exposure days (EDs). Patients with a history of inhibitor development were excluded. Participants on standard‐of‐care FVIII prophylaxis before XTEND‐1 enrolled in Arm A, where they received 52 weeks of once‐weekly efanesoctocog alfa prophylaxis (50 IU/kg). Participants receiving prior on‐demand therapy entered Arm B and received 26 weeks of on‐demand efanesoctocog alfa (50 IU/kg), followed by 26 weeks of once‐weekly efanesoctocog alfa (50 IU/kg) prophylaxis.

XTEND‐Kids (NCT04759131) included children (<12 years) with severe haemophilia A, previously treated with recombinant and/or plasma‐derived FVIII or cryoprecipitate for ≥150 EDs (patients 6 to <12 years) or >50 EDs (patients <6 years). Participants received 52 weeks of once‐weekly efanesoctocog alfa (50 IU/kg) prophylaxis.

Study protocols were approved by local Independent Ethics Committees and/or Institutional Review Boards. Studies were conducted in accordance with the International Conference on Harmonization Guidelines for Good Clinical Practice and the Declaration of Helsinki; participants or their legally authorised representative provided informed consent (Video S1).

### 2.2 Major Surgery Subgroups

Participants who underwent major surgery after the first dose of the study drug were included in the surgery subgroup for each study. Major surgery was defined as an invasive procedure that required opening a major body cavity, operation on a joint, removal of an organ, dental extraction of any molar teeth or ≥3 non‐molar teeth [9], operative alteration of normal anatomy, or crossing of a mesenchymal barrier. Major surgery was allowed in participants with ≥6 EDs of efanesoctocog alfa and a negative inhibitor test within 4 weeks before surgery.

### 2.3 Minor Surgery

Data are reported for minor surgeries where available. Minor surgery was defined as any invasive procedure in which only skin, mucous membranes, or superficial connective tissue was manipulated.

### 2.4 Dosing for Perioperative Management

Patients undergoing major surgery received a preoperative loading dose of efanesoctocog alfa 50 IU/kg. If needed, doses were adjusted for an FVIII activity level of at least 100% and maintained according to WFH guidelines [1]. Postoperatively, FVIII activity levels were maintained per WFH guidelines with doses of 30 or 50 IU/kg administered every 2–3 days, as needed. Those undergoing minor surgery received a single preoperative dose of efanesoctocog alfa (50 IU/kg). The investigator/surgeon determined perioperative dosing. Short‐term thromboembolic prophylaxis during immobilisation and/or perioperatively was allowed. The use of antifibrinolytics was permitted.

FVIII activity samples were collected according to local procedure and as required to monitor the participants’ FVIII levels during surgery and postsurgery until hospital discharge. Samples were analysed locally, but sites were requested to send an aliquot of plasma from any FVIII activity samples to the central laboratory. At least one FVIII activity sample was required to be taken each day during hospitalisation. At the central laboratory, factor activity levels were measured by activated partial thromboplastin time‐based one‐stage clotting assay with Actin FSL.

### 2.5 Surgery Endpoints and Analysis

For major surgery, secondary endpoints included the number and dose of efanesoctocog alfa injections, surgeon's/investigator's assessment of haemostatic response (based on the International Society on Thrombosis and Haemostasis 4‐point surgical procedures response scale [excellent/good/fair/poor]) [10], total factor consumption, estimated blood loss, and number and type of blood transfusions. Safety is reported for the surgical/rehabilitation period, defined as the period beginning with the preoperative loading dose of efanesoctocog alfa and ending with the latest date of either hospital discharge; perioperative follow‐up phone call; or end of 2‐week period (1‐week for minor surgery) after resuming presurgery treatment regimen. The perioperative period could be extended at investigator discretion. Emergency surgery conducted with another FVIII replacement therapy and surgery reported after the last dose of efanesoctocog alfa were not included in the analysis. Descriptive statistics are presented for major and minor surgeries.

## 3 Results

### 3.1 XTEND‐1

#### 3.1.1 Major Surgery

XTEND‐1 had 12 evaluable major surgeries (6 orthopaedic) in 11 male participants. Ten participants were in Arm A, and one was in Arm B undergoing major surgery during the prophylaxis treatment period (Table 1). Median (range) age was 47.0 (12–64) years. One participant underwent two major surgeries (hip replacement [Day 37 of the treatment period] and ulnar nerve neurolysis [Day 164]). One surgery was on a target joint (knee replacement).

**TABLE 1:** Major surgery subgroup baseline characteristics.

	XTEND‐1^a^			XTEND‐Kids
	Non‐orthopaedic (*n* = 6)	Orthopaedic (*n* = 6)	Overall (*N* = 11)	Overall (non‐orthopaedic) (*N* = 2)
**Age (years)**				
Mean (SD)	45.0 (11.3)	43.7 (16.3)	44.1 (14.0)	4.5 (0.7)
Median (range)	43.0 (33–64)	47.0 (12–56)	47.0 (12–64)	4.5 (4–5)
**Sex, *n* (%)**				
Male	6 (100)	6 (100)	11 (100)	2 (100)
Female	0	0	0	0
**Race, *n* (%)**				
American Indian or Alaska Native	0	0	0	0
Asian	1 (16.7)	2 (33.3)	3 (27.3)	0
Black or African American	0	0	0	0
White	4 (66.7)	3 (50.0)	6 (54.6)	2 (100)
Native Hawaiian or Pacific Islander	0	0	0	N/A
Other	0	0	0	0
Not reported	1 (16.7)	1 (16.7)	2 (18.2)	0
**Region, *n* (%)**				
Asia/Pacific	2 (33.3)	2 (33.3)	4 (36.4)	1 (50.0)
Europe	2 (33.3)	3 (50.0)	4 (36.4)	0
North America	1 (16.7)	1 (16.7)	2 (18.2)	1 (50.0)
South America	1 (16.7)	0	1 (9.1)	0
**Body mass index (kg/m^2^)**				
Mean (SD)	25.1 (1.4)	26.6 (1.2)	25.9 (1.5)	16.7 (1.5)
**Family history of inhibitors, *n* (%)**				
Yes	0	0	0	2 (100)
No	6 (100)	6 (100)	11 (100)	0
**Treatment arm**				
Arm A	5 (83.3)	6 (100)	10 (90.9)	N/A
Arm B^b^	1 (16.7)	0	1 (9.1)	N/A
**Target joints at baseline**				
None present	4 (66.7)	5 (83.3)	8 (72.7)	2 (100)
≥1 present	2 (33.3)	1 (16.7)	3 (27.3)	0

Abbreviations: N/A, not applicable; SD, standard deviation.

^a^ One participant underwent two major surgeries, one orthopaedic and one non‐orthopaedic.

^b^ Participant in Arm B was receiving prophylaxis at the time of surgery.

The mean (standard deviation [SD]) duration of surgery was 1.3 (0.9) h. Median (range) duration of hospitalisation was 3 (1–7) days overall, and 1 (1–3) and 5 (2–7) days for non‐orthopaedic and orthopaedic procedures, respectively.

#### 3.1.2 Perioperative Management and Haemostatic Response

**FIGURE 1:**
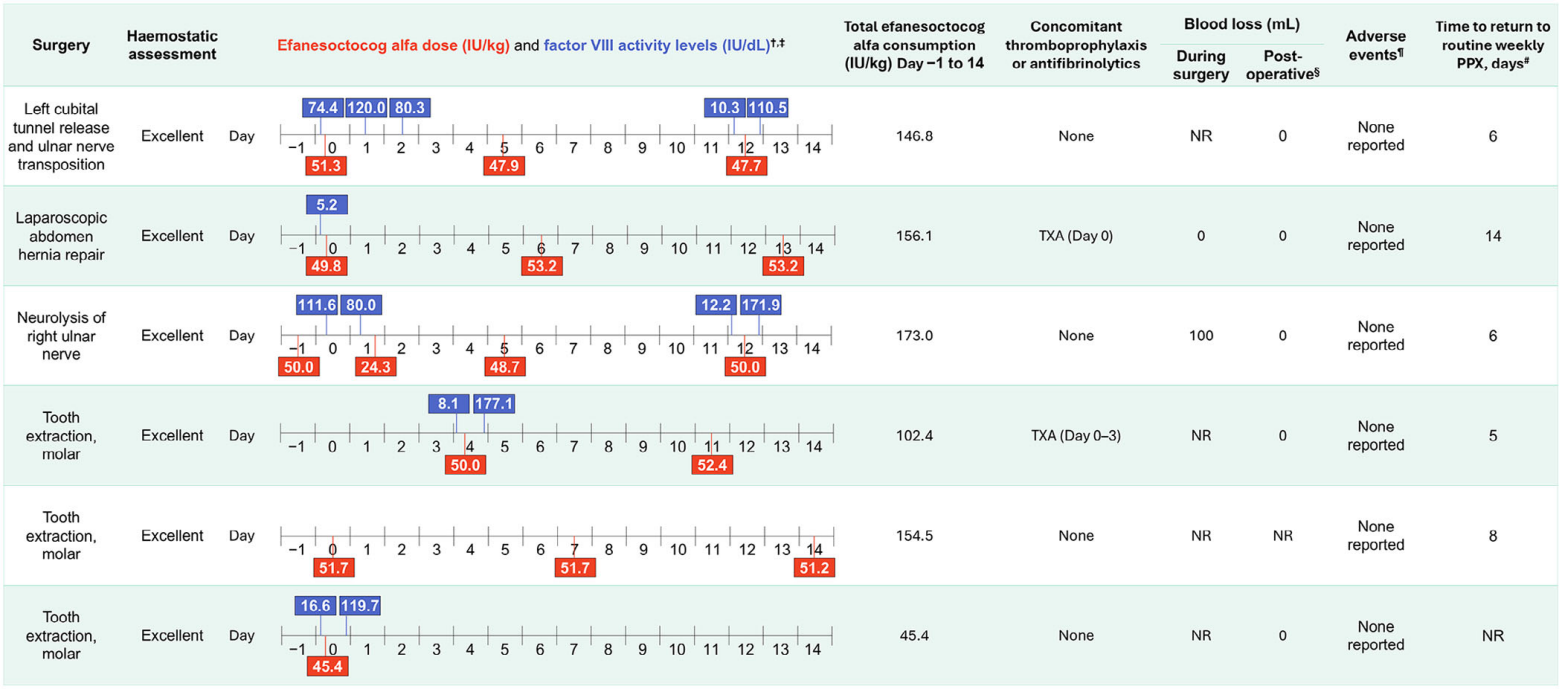
Major non‐orthopaedic surgeries during XTEND‐1. ^†^Day 0 was the day of surgery. ^‡^FVIII levels assessed at the central laboratory by aPTT‐based one‐stage clotting assay using Actin FSL. ^§^Postoperative refers to the day following the end of surgery to the date of hospital discharge. ^¶^During surgical/rehabilitation period, defined as the period beginning with a preoperative loading dose and ending with the latest of hospital discharge; perioperative follow‐up phone call; or end of 2‐week period after the resumption of presurgery treatment regimen. ^#^Calculated as: date of return to PPX − Surgery start date + 1. aPTT, activated partial thromboplastin time; FVIII, factor VIII; NR, not recorded; PPX, prophylaxis; TXA, tranexamic acid.

**FIGURE 2:**
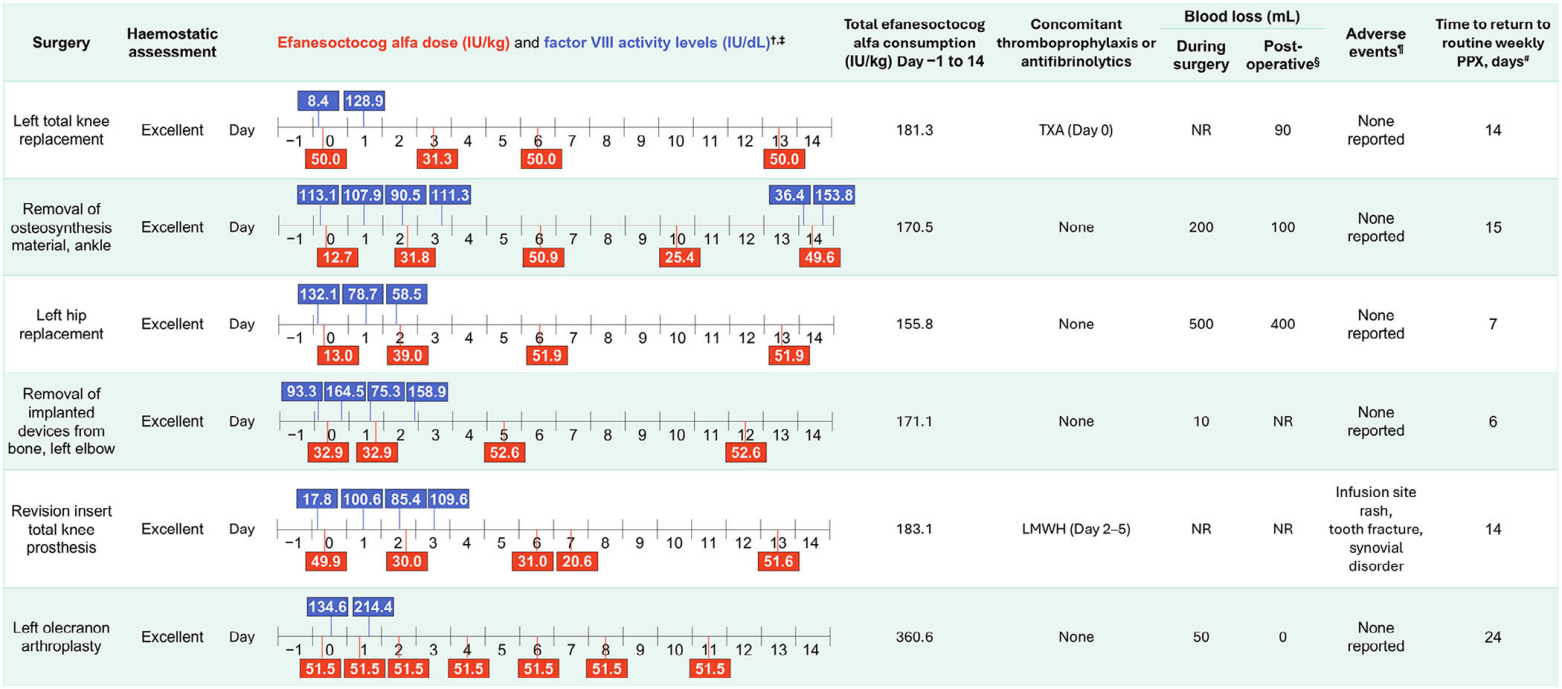
Major orthopaedic surgeries during XTEND‐1. ^†^Day 0 was the day of surgery. ^‡^FVIII levels assessed at the central laboratory by aPTT‐based one‐stage clotting assay using Actin FSL. ^§^Postoperative refers to the day following the end of surgery to the date of hospital discharge. ^¶^During surgical/rehabilitation period, defined as the period beginning with a preoperative loading dose and ending with the latest of hospital discharge; perioperative follow‐up phone call; or end of 2‐week period after the resumption of presurgery treatment regimen. ^#^Calculated as: date of return to PPX − Surgery start date + 1. aPTT, activated partial thromboplastin time; FVIII, factor VIII; LMWH, low molecular weight heparin; NR, not recorded; PPX, prophylaxis; TXA, tranexamic acid.

Individual surgical procedures are presented in Figure 1 (non‐orthopaedic) and Figure 2 (orthopaedic). FVIII levels measured on the day of surgery before the preoperative loading dose were between 5.2 and 132.1 IU/dL (eight surgeries with available data; Figures 1 and 2). FVIII levels measured on the day of surgery after the preoperative loading dose (administered Day 0 or Day −1) were between 111.6 and 164.5 IU/dL (four surgeries with available data).

For five out of six non‐orthopaedic surgeries, participants received a single preoperative injection of efanesoctocog alfa on either the day before (Day −1) or day of surgery (Day 0), with a median (range) dose per injection among treated surgeries of 50.0 (45–52) IU/kg (Table 2); for 1 non‐orthopaedic surgery (tooth extraction, molar) no preoperative efanesoctocog alfa dose was reported, and the prior dose was routine prophylaxis 3 days before surgery.

**TABLE 2:** Dosing frequency and consumption for major surgeries during XTEND‐1.

	Day −1/Day 0^a^	Days 1–3	Days 4–14	Day −1 to Day 14
**All major surgeries (*N* = 12)**				
**Number of injections**	**Number of surgeries, *n* (%)**			
0	1 (8)	5 (42)	1 (8)	0
1	11 (92)	6 (50)	0	1 (8)
2	0	1 (8)	8 (67)	1 (8)
3	0	0	2 (17)	3 (25)
4	0	0	1 (8)	4 (33)
5	0	0	0	2 (33)
6	0	0	0	0
7	0	0	0	1 (17)
**Dose per injection per surgery (IU/kg) (only among treated surgeries)**				
*n*	11	7	11	12
Mean (SD)	41.6 (15.2)	34.4 (8.7)	48.7 (5.7)	45.1 (6.2)
Median (range)	49.9 (13–52)	31.8 (24–52)	51.2 (34–53)	45.3 (34–52)
**Dose per injection per surgery (IU/kg) (inclusive of surgeries without injections during the specified perioperative period)**				
Mean (SD)	38.2 (18.8)	20.1 (18.8)	44.6 (15.1)	45.1 (6.2)
Median (range)	49.8 (0–51.7)	27.1 (0–51.5)	50.6 (0–53.2)	45.3 (34.1–52.0)
**Total consumption per surgery (IU/kg) (only among treated surgeries)**				
*n*	11	7	11	12
Mean (SD)	41.6 (15.2)	41.7 (27.4)	113.7 (31.6)	166.7 (72.5)
Median (range)	49.9 (13–52)	31.8 (24–103)	103.2 (96–206)	163.3 (45–361)
**Total consumption per surgery (IU/kg) (inclusive of surgeries without injections during the specified perioperative period)**				
Mean (SD)	38.2 (18.8)	24.4 (29.5)	104.2 (44.6)	166.7 (72.5)
Median (range)	49.8 (0–51.7)	27.1 (0–103.0)	103.0 (0–206.1)	163.3 (45.4–360.6)
Non‐orthopaedic surgeries (*n* = 6)				
Number of injections	Number of surgeries, *n* (%)			
0	1 (17)	5 (83)	1 (17)	0
1	5 (83)	1 (17)	0	1 (17)
2	0	0	5 (83)	1 (17)
3	0	0	0	3 (50)
4	0	0	0	1 (17)
**Mean dose per injection per surgery (IU/kg) (only among treated surgeries)**				
Mean (SD)	49.6 (2.5)	24.3 (N/A)	50.6 (2.1)	48.7 (3.6)
Median (range)	50.0 (45–52)	24.3 (N/A)	51.2 (48–53)	50.1 (43–52)
**Mean dose per injection per surgery (IU/kg) (inclusive of surgeries without injections during the specified perioperative period)**				
Mean (SD)	41.3 (20.4)	4.1 (9.9)	42.2 (20.7)	48.7 (3.6)
Median (range)	49.9 (0–52)	0.0 (0–24)	50.3 (0–53)	50.1 (43–52)
**Total consumption per surgery (IU/kg) (only among treated surgeries)**				
Mean (SD)	49.6 (2.5)	24.3 (N/A)	101.2 (4.2)	129.7 (47.6)
Median (range)	50.0 (45–52)	24.3 (N/A)	102.4 (96–106)	150.7 (45–173)
**Total consumption per surgery (IU/kg) (inclusive of surgeries without injections during the specified perioperative period)**				
Mean (SD)	41.3 (20.4)	4.1 (9.9)	84.3 (41.5)	129.7 (47.6)
Median (range)	49.9 (0–52)	0.0 (0–24)	100.5 (0–106)	150.7 (45–173)
**Orthopaedic surgeries (*n* = 6)**				
**Number of injections**	**Number of surgeries, *n* (%)**			
0	0	0	0	0
1	6 (100)	5 (83)	0	0
2	0	1 (17)	3 (50)	0
3	0	0	2 (33)	0
4	0	0	1 (17)	3 (50)
5	0	0	0	2 (33)
6	0	0	0	0
7	0	0	0	1 (17)
**Mean dose per injection**				
Mean (SD)	35.0 (18.5)	36.1 (8.2)	47.1 (7.3)	41.5 (6.4)
Median (range)	41.4 (13–52)	32.4 (30–52)	50.8 (34–53)	40.9 (34–52)
**Total consumption (IU/kg)**				
Mean (SD)	35.0 (18.5)	44.7 (28.8)	124.1 (41.2)	203.7 (77.5)
Median (range)	41.4 (13–52)	32.4 (30–103)	104.6 (100–206)	176.2 (156–361)

Abbreviations: N/A, not applicable; SD, standard deviation.

^a^ Day 0 was the day of surgery.

For orthopaedic surgeries, all six patients received a single injection of efanesoctocog alfa on Day 0, with a median (range) dose of 41.4 (13–52) IU/kg (Table 2); 2 patients received a single routine prophylaxis dose on Day −1, and a lower preoperative dose of ∼13 IU/kg on Day 0. Haemostatic response on Day 0 was rated as excellent for all surgeries.

During Days 1–3 postsurgery, no efanesoctocog alfa dose was given for 5 of 12 major surgeries (all non‐orthopaedic), with the remaining receiving either 1 (*n* = 6) or 2 doses (*n* = 1) (Table 2). The median (range) total number of doses that were given during Day −1 to Day 14 was 4 (1–7). Median (range) total consumption during Day −1 to Day 14 (including routine prophylaxis) for non‐orthopaedic and orthopaedic surgeries was 150.7 (45–173) IU/kg among treated surgeries and 176.2 (156–361) IU/kg, respectively. The median (range) time to return to routine prophylaxis postsurgery was 10 (4–23) days.

Thromboprophylaxis (low molecular weight heparin) was administered to only 1 patient (>50 years old; revision insert total knee prosthesis) on Days 2–5 postsurgery. Antifibrinolytic therapy (tranexamic acid) was administered to three patients (Figures 1 and 2).

#### 3.1.3 Blood Loss and Blood Transfusion

For major surgeries with available data, the median (range) estimated blood loss was 75 (0–500) mL during surgery (*n* = 6). Median (range) estimated postoperative blood loss was 0 (0–400) mL (*n* = 9). No participants required blood transfusions.

#### 3.1.4 Safety During Major Surgery

One participant experienced three treatment‐emergent adverse events (TEAEs) during the surgical/rehabilitation period (infusion site rash, tooth fracture, and synovial disorder [thickened synovium left knee]; Figure 2). These TEAEs were classified as mild, nonserious, and not related to efanesoctocog alfa as assessed by the Investigator. No thromboembolic complications or inhibitor development was reported.

#### 3.1.5 Minor Surgery

Fifteen participants underwent 18 minor surgeries during XTEND‐1; procedures included dental implant surgery (*n* = 4), root canal (*n* = 3), non‐molar dental extraction (*n* = 3), endoscopy/colonoscopy (*n* = 3), cataracts (n = 2), nasal basal cell carcinoma excision and local flap (*n* = 1), cecostomy tube change (*n* = 1), and port‐a‐cath removal (*n* = 1). For 13 minor surgeries in 12 participants, a single dose of efanesoctocog alfa was received on Day 0, with a median (range) dose of 51.2 (48–52) IU/kg. For five minor surgeries (all dental), participants received no preoperative dose of efanesoctocog alfa. Haemostatic response was rated excellent for all minor surgeries assessed (*n* = 13). Blood loss during surgery of 10 mL was reported for the port‐a‐cath removal procedure.

During Days 1–3, four participants received one injection of efanesoctocog alfa (median [range] dose per injection among treated surgeries: 40.9 [29–52] IU/kg) (Table 3). During Days 4–7, 10 participants had injections (median [range] dose per injection among treated surgeries: 51.2 [50–52] IU/kg). Median (range) total consumption among treated minor surgeries during Day −1 to Day 7 (including routine prophylaxis) was 52.5 (50–135.8) IU/kg (mean [SD] 78.9 [31.8] IU/kg). For one minor surgery during the on‐demand treatment period, one (joint) bleeding event was reported that was unrelated to surgery during Days 4–7. Perioperative antifibrinolytic therapy (tranexamic acid) was administered to four patients undergoing minor surgery.

**TABLE 3:** Dosing frequency and consumption for minor surgeries during XTEND‐1.

	Day −1/Day 0^a^	Days 1–3	Days 4–7	Day −1 to Day 7
**All minor surgeries (*N* = 18)**				
**Dose per injection per surgery (IU/kg) (among treated surgeries)**				
*n*	13	4	10	17
Mean (SD)	51.2 (1.2)	40.8 (12.2)	51.3 (0.8)	50.4 (2.6)
Median (range)	51.2 (48–52)	40.9 (29–52)	51.2 (50–52)	51.1 (43–52)
**Dose per injection per surgery (IU/kg) (inclusive of surgeries without injections during the specified perioperative period)**				
Mean (SD)	37.0 (23.6)	9.1 (18.2)	28.5 (26.2)	47.6 (12.1)
Median (range)	50.9 (0–52)	0.0 (0–52)	50.3 (0–52)	50.9 (0–52)
**Total consumption per surgery (IU/kg) (among treated surgeries)**				
*n*	13	4	10	17
Mean (SD)	51.2 (1.2)	40.8 (12.2)	51.3 (0.8)	78.9 (31.8)
Median (range)	51.2 (48–52)	40.9 (29–52)	51.2 (50–52)	52.5 (50–136)
**Total consumption per surgery (IU/kg) (inclusive of surgeries without injections during the specified perioperative period)**				
Mean (SD)	37.0 (23.6)	9.1 (18.2)	28.5 (26.2)	74.5 (36.0)
Median (range)	50.9 (0–52)	0.0 (0–52)	50.3 (0–52)	52.5 (0–136)

*Note*: Mean dose per injection is the average dose across all injections per minor surgery (including loading dose). Total consumption is summarised over all injections during the referenced time period in the surgical/rehabilitation period.

Abbreviation: SD, standard deviation.

^a^ Day 0 was the day of surgery.

No thromboembolic complications or inhibitor development were reported.

### 3.2 XTEND‐Kids

#### 3.2.1 Major Surgery

Two participants (4 and 5 years of age) underwent one major surgery during XTEND‐Kids (Table 1). The surgeries were extensive dental restoration requiring general anaesthesia (including one tooth extraction) and circumcision. The duration of surgery was 1.5 and 0.6 h, respectively. Hospital discharge was the same day as surgery for the dental procedure and the day after surgery for the circumcision.

#### 3.2.2 Perioperative Management and Haemostatic Response

Each surgery had one preoperative loading dose of 61.9 and 60.4 IU/kg. Predose FVIII activity level on Day 0 was 32.7 IU/dL for one participant (dental surgery) and 59.5 IU/dL for the other (circumcision). Haemostatic response on Day 0 was rated as excellent for both. In the participant with dental surgery, a dose of 37.1 IU/kg was administered on Day 2, and he resumed routine weekly prophylaxis from Day 4. The participant who underwent circumcision resumed once‐weekly prophylactic dosing on Day 6. Tranexamic acid was administered on Day −1 to the patient who underwent dental surgery and administered on Day 0 to Day 5 postsurgery to the patient who underwent circumcision.

#### 3.2.3 Blood Loss and Blood Transfusion

Estimated blood loss was 0 mL during the dental surgery and postsurgery. During the circumcision, the estimated blood loss was 50 mL; postoperative blood loss of 10 mL was reported on Day 0. Neither required blood transfusion.

#### 3.2.4 Safety During Major Surgery

One TEAE unrelated to efanesoctocog alfa was reported for each participant between Day 0 and Day 14 of the surgical/rehabilitation period. The participant who underwent dental surgery experienced a viral rash over the upper lip. The surgery of circumcision was reported as a severe, serious TEAE, due to hospitalisation for the procedure. No thromboembolic complications or inhibitor development was reported.

#### 3.2.5 Minor Surgery

Eight participants underwent nine minor surgeries during XTEND‐Kids, including port replacement (*n* = 2), port removal (*n* = 1), esophagogastroduodenoscopy (*n* = 1), dental extraction (*n* = 2), port revision (*n* = 1), and gastroscopy examinations (*n* = 2). For all minor surgeries, a single preoperative loading dose of efanesoctocog alfa of median (range) 53.5 (39–59) IU/kg was administered (Day 0: *n* = 7; Day −1: *n* = 2). Haemostatic response was rated excellent for all minor surgeries assessed (*n* = 8). Median (range) blood loss during surgery was 0 (0–15) mL and postoperatively was 0 (0–5) mL. During Days 1–3, one participant had one injection and one participant had two injections (median [range] dose per injection among treated surgeries: 46.8 [34–59] IU/kg; median [range] dose per injection inclusive of surgeries not treated: 0.0 [0–59] IU/kg). Most patients (6 of 8) who underwent minor surgeries did not receive additional dosing during the postoperative period. Median (range) total consumption during Day −1 to Day 7 (including routine prophylaxis) was 107.3 (48–199) IU/kg. No patients required blood transfusions; none experienced postoperative bleeding episodes. Antifibrinolytic therapy was administered to two patients (tranexamic acid [*n* = 1]; aminocaproic acid [*n* = 1]) on Day 0 and to one patient 5 days postsurgery.

Three TEAEs were reported in three participants during Days 0–7 of the surgical/rehabilitation period (catheter site pain, suspected eosinophilic oesophagitis, and device [port‐a‐cath] malfunction), of which two were serious (suspected eosinophilic oesophagitis, device malfunction). TEAEs were assessed as unrelated to efanesoctocog alfa treatment. No thromboembolic complications or FVIII inhibitors were reported.

## 4 Discussion

Efanesoctocog alfa was effective and well tolerated for perioperative management of adults/adolescents and children with severe haemophilia A during major and minor surgeries in XTEND‐1 and XTEND‐Kids. Efanesoctocog alfa maintained haemostasis on the day of surgery with either a single or no preoperative dose; haemostatic response was rated as excellent. Perioperative consumption of efanesoctocog alfa and injection frequency were low and comparable to that expected for routine prophylaxis in the absence of surgery. No safety concerns were identified.

Consumption of efanesoctocog alfa in adults/adolescents on the day of major surgery (including preoperative dose on Day 0/Day −1) was lower than consumption reported for SHL and EHL products, which often require multiple injections on surgery days [11, 12, 13, 14, 15, 16]. For example, during the PROTECT VIII trial, damoctocog alfa pegol (EHL) injections on the day of major surgery ranged from 1 to 3, with over half (16/26) of surgeries requiring more than one dose [13]. Consumption of efanesoctocog alfa for major surgeries was also comparable to consumption during routine efanesoctocog alfa prophylaxis. WFH guidelines recommend maintaining factor activity levels within target ranges during surgery; the substantial improvement in FVIII half‐life and AUC provided by efanesoctocog alfa enables maintenance of FVIII activity levels with less frequent administration of lower doses and potentially less frequent perioperative measurement of plasma FVIII levels, lowering treatment and monitoring burden [5, 6]. In some cases, preoperative doses of efanesoctocog alfa were not required, as factor levels under routine prophylaxis were judged to be sufficient to provide adequate haemostatic coverage for surgery.

Effective bleed protection provided by efanesoctocog alfa facilitated early postsurgery discharge. Duration of hospitalisation for major surgery was short (median [range]: 3 [1–7] days); patients returned to routine prophylaxis quickly postsurgery (median [range]: 10 [4–23] days). Five of the 12 major surgeries in XTEND‐1 (all non‐orthopaedic) required no dose of efanesoctocog alfa during Days 1–3 postsurgery. During Day −1 to Day 14, the median total consumption for major surgery was 163.3 IU/kg, with consumption higher for orthopaedic (176.2 IU/kg) than non‐orthopaedic surgeries (150.7 IU/kg). In comparison, in the Phase 3 trial of rurioctocog alfa pegol (EHL), the median (range) total dose of rurioctocog alfa pegol per patient was 489 IU/kg for major non‐orthopaedic surgeries and 629 IU/kg for major orthopaedic surgeries [11].

Efanesoctocog alfa also provided efficient protection for minor surgeries in patients of all ages. Five minor dental surgeries during prophylaxis in adults/adolescents required no preoperative dose of efanesoctocog alfa; all others required only a single preoperative dose of ∼50 IU/kg to maintain haemostasis on the day of surgery, with haemostatic response rated as excellent. Regular efanesoctocog alfa prophylaxis or a single additional preoperative dose provided effective management for minor surgeries, which may be more efficient compared with other FVIII replacement products [16, 17], though data on surgery in children with other FVIII products is sparse. The high‐sustained FVIII activity provided by efanesoctocog alfa may reduce the requirement of additional doses for minor surgeries between the weekly prophylaxis dosing interval.

Blood loss reported during surgeries was as expected for a patient without haemophilia; no blood transfusions were required. No safety concerns were reported. All TEAEs during surgery were unrelated to efanesoctocog alfa. Intensive treatment with factor replacement therapies, which is often required during perioperative management, is associated with inhibitor development (mainly in mild haemophilia) [18]. Efanesoctocog alfa enables less intensive treatment for patients undergoing surgery. No thromboembolic complications or inhibitor development was reported in XTEND‐1 or XTEND‐Kids.

Due to the heterogeneity of surgical procedures, variation in surgical management at different treatment centres, and small sample sizes, it is difficult to compare products for haemostatic efficacy during perioperative management. A limitation of this analysis is the sample size, although it met minimum regulatory requirements for assessing efficacy for perioperative management [19]. Another limitation is the variation in local standard procedures for perioperative management, as the investigator/surgeon made care decisions including FVIII activity monitoring, efanesoctocog alfa dose, and injection frequency. Furthermore, no standard definitions exist for the classification of major and minor surgery in people with haemophilia [9]. Some procedures, such as circumcision, have been classified as minor and major surgeries in different studies [9]. Despite these limitations, the surgeries in these studies were varied and reflected common surgeries in the haemophilia population.

## 5 Conclusion

Results from surgeries during XTEND‐1 and XTEND‐Kids demonstrate that efanesoctocog alfa provided simple and highly effective perioperative management of both adults/adolescents and children with severe haemophilia A, with low injection frequency, low consumption, and short hospitalisation times.

## Ethics Statement

The study was performed in accordance with the Declaration of Helsinki and local regulations. The protocols were approved by institutional review boards and/or ethics committees at participating institutes.

## Consent

Written informed consent from participants or their legally authorised representative was obtained by investigative sites before their enrolment in the study. A statement of informed consent was obtained meeting the requirements of 21 Code of Federal Regulations 50, local regulations, International Council for Harmonisation of Technical Requirements for Pharmaceuticals for Human Use (ICH) guidelines, privacy and data protection requirements including those of the Global Data Protection Regulation (GDPR), Health Insurance Portability and Accountability Act (HIPAA) requirements, where applicable, and the institutional review board/independent ethics committee or study centre.

## Conflicts of Interest

Robert Klamroth has received honoraria and/or been a member of advisory committees for Bayer, Biomarin, Biotest, CSL Behring, Grifols, Novo Nordisk, Octapharma, Pfizer, Roche/Chugai, Sanofi, Sobi, and Shire/Takeda. Annette von Drygalski has received fees from Biomarin, Bioverativ/Sanofi‐Genzyme, Novo Nordisk, Takeda, and Uniqure for participation in industry‐sponsored education events and advisory boards. She has received research funding from Bioverativ/Sanofi‐Genzyme and Pfizer. She is a co‐founder and a member of the board of directors of Hematherix Inc. She holds a patent for a superFactorVa, and she is the inventor and physician lead for the Joint Activity and Damage Examination (JADE) ultrasound measurement tool. JADE is copyrighted through the Office of Innovation and Commercialization at the University of California, San Diego. Cedric Hermans has received consultancy and/or lecture fees from Bayer, Shire/Takeda, Roche, CSL Behring, Novo Nordisk, Octapharma, Sanofi, Pfizer, Sobi, LFB, Octapharma, Uniqure, CSL Behring, and Biomarin. Young‐Shil Park reports research support or participation as a principal investigator from BioMarin Pharmaceutical Inc., CSL Behring, Novo Nordisk, Sanofi, Takeda, Pfizer, and Chugai. Anthony K. C. Chan has received research support from Bayer, Novo Nordisk, Pfizer, Sanofi, and Takeda. He also reports participation in scientific advisory boards and honoraria from Bayer, Novo Nordisk, Octapharma, Pfizer, Sanofi, and Takeda. Alphan Kupesiz reports clinical trials for Bayer, Novo Nordisk, Pfizer, Sanofi, and Roche; and advisory board membership for Novo Nordisk, Roche, Pfizer, Sobi, and Takeda. María Teresa Alvarez‐Román has received consultancy and/or lecture fees from Bayer, Takeda, Roche, CSL Behring, Novo Nordisk, Octapharma, Sanofi, Pfizer, Sobi, LFB, and Biomarin. Lynn Malec has received consultancy fees from Bayer, Sanofi Genzyme, Sobi, Genentech, Biomarin, CSL Behring, Spark Therapeutics, and Novo Nordisk. She has received research funding from Sanofi Genzyme. Elena Santagostino, Linda Bystrická, and Lydia Abad‐Franch are employees of Sobi and may hold shares and/or stock options in the company. Jennifer Dumont, Graham Neill, and Lila‐Sabrina Fetita are employees of Sanofi and may hold shares and/or stock options in the company. Liane Khoo received fees from Biomarin, Bioverativ/Sanofi‐Genzyme, and Roche for participation in industry‐sponsored education events and advisory boards.

## Supporting information



Supporting Information

## Data Availability

Qualified researchers may request access to patient‐level data and related documents (including, e.g., the clinical study report, study protocol with any amendments, blank case report form, statistical analysis plan, and dataset specifications). Patient‐level data will be anonymised, and study documents will be redacted, including to protect the privacy of trial participants. Further details on Sanofi's data sharing criteria, eligible studies, and process for requesting access can be found at https://www.vivli.com.
